# Influence of passive hyperthermia and diurnal variation on exercise performance and cognitive function in the heat

**DOI:** 10.1186/2046-7648-4-S1-A155

**Published:** 2015-09-14

**Authors:** Hidenori Otani, Mitsuharu Kaya, Akira Tamaki, Heita Goto, Junzo Tsujita

**Affiliations:** 1 Himeji Dokkyo University, Himeji, Japan; 2 Hyogo University of Health, Kobe, Japan; 3 Kyushu Kyoritsu University, Kitakyushu, Japan; 4 Hyogo College of Medicine, Nishinomiya, Japan

## Introduction

Both aerobic and anaerobic exercise performances have a diurnal variation. As commonly reported in previous studies, circadian rhythm in exercise performance is low in the morning and peaks in the evening. It has been demonstrated that hyperthermia before exercise attenuates subsequent exercise performance in the heat. However, combined effects of passive hyperthermia and the time-of-day on both aerobic and anaerobic exercise capacity and cognitive function in the heat have not been evaluated. Therefore, the aim of this study was to examine the effects of passive hyperthermia and circadian rhythm on aerobic and anaerobic exercise performances and cognitive function after exercise in the heat.

## Methods

Eight male volunteers completed four trials which involved anaerobic and aerobic cycling performance tests in a climatic chamber (30°C, 50% rh) at two different times-of-day: 08:00 (morning) and 17:00 (evening) h. The anaerobic performance test consisted of a 10 sec maximal sprint at 5 kp to determine the maximal anaerobic power. The aerobic performance test consisted of cycling at 60% maximum oxygen uptake until exhaustion to determine exercise time to exhaustion. Participants cycled after a 30 min seated rest in the morning (AR) and evening (PR), and a 30 min water immersion at 40°C to the upper chest in the morning (AH) and evening (PH) to induce hyperthermia at core temperature of about 38°C. Experimental trials were completed in a randomised order. Rectal temperature (Tre), skin temperature (chest, upper arm, thigh and calf), heart rate, skin blood flow and blood pressure were recorded. The cognitive function test after exhaustion involved the completion of two computer-based tests which included the Stroop and Sternburg tests. Data are presented as mean (SD). Data collected once a trial were analysed using a one-way repeated measures ANOVA. Data collected over time were analysed using a two-way (trial-by-time) repeated measures ANOVA. Pair-wise differences between the trials were evaluated using one-way ANOVAs with a Bonferroni adjustment applied for multiple comparisons.

## Results

Tre at the start of exercise was higher in AH and PH than in AR and PR (AR 36.8[0.4] °C; AH 37.9[0.2] °C; PR 37.3[0.3] °C; PH 38.0[0.2] °C; p < 0.0001). Maximal anaerobic power was not different between the trials (AR 11.5[1.7] W.kg^-1^; AH 12.5[1.9] W.kg^-1^; PR 11.7[1.4] W.kg^-1^; PH 12.0[1.9] W.kg^-1^; p = 0.24). Exercise time to exhaustion was reduced in AH (15[8] min) and PH (24[9] min) compared to AR (39[16] min; p < 0.05), and in AH compared to PR (43[24] min; p < 0.05). At the point of exhaustion, Tre, mean skin temperature, heart rate and cutaneous vascular conductance were not different between the trials. Both cognitive function tests were not different between the trials. However, in the Sternberg test, response time in the three letters test was longer and errors in the five letters test was larger during the hyperthermia trials (AH and PH) than in the seated rest trials (AR and PR) (P < 0.05).

## Discussion

In this study, passive hyperthermia before exercise significantly attenuated aerobic exercise capacity only in the morning. This result may indicate that high core temperature at the start of exercise have a deteriorate effect on aerobic exercise performance in the heat in the morning. In addition, passive hyperthermia before exercise significantly impaired the results of the Sternberg test, implying that hyperthermia attenuates cognitive function.

## Conclusion

This study demonstrates that passive hyperthermia before exercise elicits significant reductions in aerobic exercise performance in the heat in the morning, but not in the evening. Hyperthermia and circadian rhythm, however, do not influence anaerobic exercise performance in the heat. Moreover, hyperthermia impairs cognitive function, and diurnal variation does not cause such effect.

